# Advances in Multi-Sensor Data Fusion: Algorithms and Applications

**DOI:** 10.3390/s91007771

**Published:** 2009-09-30

**Authors:** Jiang Dong, Dafang Zhuang, Yaohuan Huang, Jingying Fu

**Affiliations:** Data Center for Resources and Environmental Sciences, State Key Lab of Resources and Environmental Information System, Institute of Geographical Sciences and Natural Resources Research, Chinese Academy of Sciences, Beijing 100101, China; E-Mails: zhuangdf@igsnrr.ac.cn (D.Z.); huangyh@lreis.ac.cn (Y.H.); fujingying.2005@163.com (J.F.)

**Keywords:** multi-sensor, data fusion, remote sensing

## Abstract

With the development of satellite and remote sensing techniques, more and more image data from airborne/satellite sensors have become available. Multi-sensor image fusion seeks to combine information from different images to obtain more inferences than can be derived from a single sensor. In image-based application fields, image fusion has emerged as a promising research area since the end of the last century. The paper presents an overview of recent advances in multi-sensor satellite image fusion. Firstly, the most popular existing fusion algorithms are introduced, with emphasis on their recent improvements. Advances in main applications fields in remote sensing, including object identification, classification, change detection and maneuvering targets tracking, are described. Both advantages and limitations of those applications are then discussed. Recommendations are addressed, including: (1) Improvements of fusion algorithms; (2) Development of “algorithm fusion” methods; (3) Establishment of an automatic quality assessment scheme.

## 1. Introduction

With the development of multiple types of biosensors, chemical sensors, and remote sensors on board satellites, more and more data have become available for scientific researches. As the volume of data grows, so does the need to combine data gathered from different sources to extract the most useful information. Data fusion is an effective way for optimum utilization of large volumes of data from multiple sources. Multi-sensor data fusion seeks to combine information from multiple sensors and sources to achieve inferences that are not feasible from a single sensor or source. The fusion of information from sensors with different physical characteristics enhances the understanding of our surroundings and provides the basis for planning, decision-making, and control of autonomous and intelligent machines [1]. In the past decades it has been applied to different fields such as pattern recognition, visual enhancement, object detection and area surveillance [2].

The literature on data fusion in computer vision, machine intelligence and medical imaging is substantial, but will not be discussed here. This paper is focused on multi-sensor data fusion in the satellite remote sensing field. Remote sensing techniques have proven to be powerful tools for the monitoring of the Earth's surface and atmosphere on a global, regional, and even local scale, by providing important coverage, mapping and classification of land cover features such as vegetation, soil, water and forests [3] The volume of remote sensing images continues to grow at an enormous rate due to advances in sensor technology for both high spatial and temporal resolution systems. Consequently, an increasing quantity of image data from airborne/satellite sensors have been available, including multi-resolution images, multi-temporal images, multi-frequency/spectral bands images and multi-polarization image. Multi-sensor data fusion is a process of combining images, obtained by sensors of different wavelengths to form a composite image. The composite image is formed to improve image content and to make it easier for the user to detect, recognize, and identify targets and increase situational awareness.

In 1997, Hall and Llinas gave a general introduction to multi-sensor data fusion [1]. Another in-depth review paper on multiple sensors data fusion techniques was published in 1998 [2]. This paper explained the concepts, methods and applications of image fusion as a contribution to multi-sensor integration oriented data processing. Since then, image fusion has received increasing attention. Further scientific papers on image fusion have been published with an emphasis on improving fusion quality and finding more application areas. As a case in point, Simone *et al.* describe three typical applications of data fusion in remote sensing, such as obtaining elevation maps from synthetic aperture radar (SAR) interferometers, the fusion of multi-sensor and multi-temporal images, and the fusion of multi-frequency, multi-polarization and multi-resolution SAR images [3]. Vijayaraj provided the concepts of image fusion in remote sensing applications [4]. Quite a few survey papers have been published recently, providing overviews of the history, developments, and the current state of the art of image fusion in the image-based application fields [5-7], but recent development of multi-sensor data fusion in remote sensing fields has not been discussed in detail. The objectives of this paper are to present an overview of new advances in multi-sensor satellite image fusion, focused on its main application fields in remote sensing. The paper is organized into four sections. Section 2 describes the categorization and the advance in algorithm; Section 3 describes advance in application, such as feature extraction, classification, change detection and maneuvering targets tracking; conclusions are drawn in Section 4.

## 2. Advances in Algorithms

### 2.1. Categorization of the algorithms

**Figure 1.**
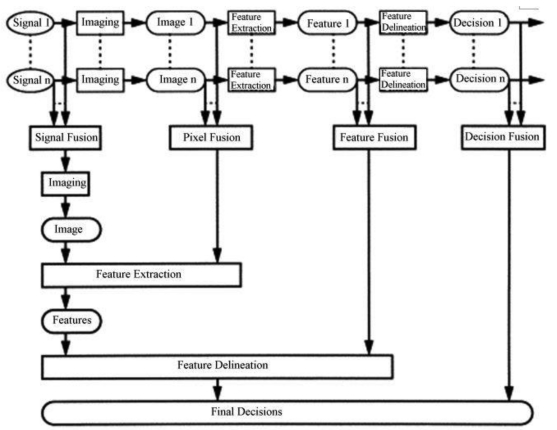
An overview of categorization of the fusion algorithms [8].

Multi-sensor data fusion can be performed at four different processing levels, according to the stage at which the fusion takes place: signal level, pixel level, feature level, and decision level. Figure 1 illustrates of the concept of the four different fusion levels [8].

1. Signal level fusion. In signal-based fusion, signals from different sensors are combined to create a new signal with a better signal-to noise ratio than the original signals.
2. Pixel level fusion. Pixel-based fusion is performed on a pixel-by-pixel basis. It generates a fused image in which information associated with each pixel is determined from a set of pixels in source images to improve the performance of image processing tasks such as segmentation
3. Feature level fusion. Feature-based fusion at feature level requires an extraction of objects recognized in the various data sources. It requires the extraction of salient features which are depending on their environment such as pixel intensities, edges or textures. These similar features from input images are fused.
4. Decision-level fusion consists of merging information at a higher level of abstraction, combines the results from multiple algorithms to yield a final fused decision. Input images are processed individually for information extraction. The obtained information is then combined applying decision rules to reinforce common interpretation.

### 2.2. Advances in fusion algorithms

Among the hundreds of variations of image fusion techniques, the most popular and effective methods include, but are not limited to, intensity-hue-saturation (IHS), high-pass filtering, principal component analysis (PCA), different arithmetic combination(e.g., Brovey transform), multi-resolution analysis-based methods (e.g., pyramid algorithm, wavelet transform), and Artificial Neural Networks (ANNs). The paper will provide a general introduction to those selected methods with emphases on new advances in the remote sensing field.

#### 2.2.1. Standard fusion algorithms

The PCA transform converts inter-correlated multi-spectral (MS) bands into a new set of uncorrelated components. To do this approach first we must get the principle components of the MS image bands. After that, the first principle component which contains the most information of the image is substituted by the panchromatic image. Finally the inverse PC transform is done to get the new RGB (Red, Green, and Blue) bands of multi-spectral image from the principle components.

The IHS fusion converts a color MS image from the RGB space into the IHS color space. Because the intensity (I) band resembles a panchromatic (PAN) image, it is replaced by a high-resolution PAN image in the fusion. A reverse IHS transform is then performed on the PAN together with the hue (H) and saturation (S) bands, resulting in an IHS fused image.

Different arithmetic combinations have been developed for image fusion. The Brovey transform, Synthetic Variable Ratio (SVR), and Ratio Enhancement (RE) techniques are some successful examples [9]. The basic procedure of the Brovey transform first multiplies each MS band by the high resolution PAN band, and then divides each product by the sum of the MS bands. The SVR and RE techniques are similar, but involve more sophisticated calculations for the MS sum for better fusion quality.

The Standard fusion algorithms mentioned above have been widely used for relatively simple and time efficient fusion schemes. However, three problems must be considered before their application: (1) Standard fusion algorithms generate a fused image from a set of pixels in the various sources. These pixel-level fusion methods are very sensitive to registration accuracy, so that co-registration of input images at sub-pixel level is required; (2) One of the main limitations of HIS and Brovey transform is that the number of input multiple spectral bands should be equal or less than three at a time; (3) Standard image fusion methods are often successful at improves the spatial resolution, however, they tend to distort the original spectral signatures to some extent [9,10]. More recently new techniques such as the wavelet transform seem to reduce the color distortion problem and to keep the statistical parameters invariable.

#### 2.2.2. Wavelet-based methods

Multi-resolution or multi-scale methods, such as pyramid transformation, have been adopted for data fusion since the early 1980s [11]. The Pyramid-based image fusion methods, including Laplacian pyramid transform, were all developed from Gaussian pyramid transform, have been modified and widely used, and substituted by the wavelet transform methods in some extend in recent years [12,13]. In 1989, Mallat put all the methods of wavelet construction into the framework of functional analysis and described the fast wavelet transform algorithm and general method of constructing wavelet orthonormal basis. On the basis, wavelet transform can be really applied to image decomposition and reconstruction [14-16].

**Figure 2.**
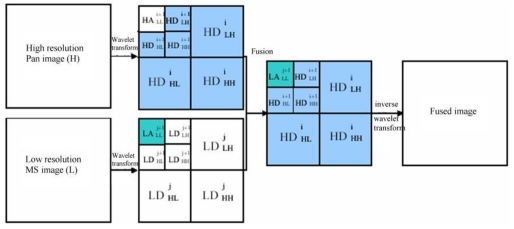
Generic flowchart of wavelet-based image fusion.

Wavelet transforms provide a framework in which an image is decomposed, with each level corresponding to a coarser resolution band. For example, in the case of fusing a MS image with a high-resolution PAN image with wavelet fusion, the Pan image is first decomposed into a set of low-resolution Pan images with corresponding wavelet coefficients (spatial details) for each level. Individual bands of the MS image then replace the low-resolution Pan at the resolution level of the original MS image. The high resolution spatial detail is injected into each MS band by performing a reverse wavelet transform on each MS band together with the corresponding wavelet coefficients (Figure 2).

In the wavelet-based fusion schemes, detail information is extracted from the PAN image using wavelet transforms and injected into the MS image. Distortion of the spectral information is minimized compared to the standard methods mentioned in Section 2.2.1 [17]. In order to achieve optimum fusion results, various wavelet-based fusion schemes had been tested by many researchers. Among these schemes several new concepts/algorithms were presented and discussed. Candes provided a method for fusing SAR and visible MS images using the Curvelet transformation. The method was proven to be more efficient for detecting edge information and denoising than wavelet transformation [18]. Curvelet-based image fusion has been used to merge a Landsat ETM+ panchromatic and multiple-spectral image. The proposed method simultaneously provides richer information in the spatial and spectral domains [19]. Donoho *et al.* presented a flexible multi-resolution, local, and directional image expansion using contour segments, the Contourlet transform, to solve the problem that wavelet transform could not efficiently represent the singularity of linear/curve in image processing [20,21]. Contourlet transform provides flexible number of directions and captures the intrinsic geometrical structure of images.

In general, as a typical feature level fusion method, wavelet-based fusion could evidently perform better than convenient methods in terms of minimizing color distortion and denoising effects. It has been one of the most popular fusion methods in remote sensing in recent years, and has been standard module in many commercial image processing soft wares, such as ENVI, PCI, ERDAS. Problems and limitations associated with them include: (1) Its computational complexity compared to the standard methods; (2) Spectral content of small objects often lost in the fused images; (3) It often requires the user to determine appropriate values for certain parameters (such as thresholds). The development of more sophisticated wavelet-based fusion algorithm (such as Ridgelet, Curvelet, and Contourlet transformation) could improve the performance results, but these new schemes may cause greater complexity in the computation and setting of parameters.

#### 2.2.3. Artificial neural network

**Figure 3.**
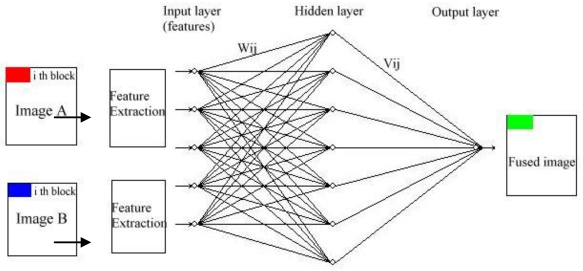
General schematic diagram of the ANN-based image fusion method.

Artificial neural networks (ANNs) have proven to be a more powerful and self-adaptive method of pattern recognition as compared to traditional linear and simple nonlinear analyses [22,23]. The ANN-based method employs a nonlinear response function that iterates many times in a special network structure in order to learn the complex functional relationship between input and output training data. The General schematic diagram of the ANN-based image fusion method can be seen in Figure 3. The input layer has several neurons, which represent the feature factors extracted and normalized from image A and image B. The hidden layer has several neurons and the output layer has one neuron (or more neuron). The *i*th neuron of the input layer connects with the *j*th neuron of the hidden layer by weight W_ij_, and weight between the jth neuron of the hidden layer and the *t*th neuron of output layer is V_jt_ (in this case *t* = 1). The weighting function is used to simulate and recognize the response relationship between features of fused image and corresponding feature from original images (image A and image B).

As the first step of ANN-based data fusion, two registered images are decomposed into several blocks with size of M and N (Figure 3). Then, features of the corresponding blocks in the two original images are extracted, and the normalized feature vector incident to neural networks can be constructed [24]. The features used here to evaluate the fusion effect are normally spatial frequency, visibility, and edge. The next step is to select some vector samples to train neural networks. An ANN is a universal function approximator that directly adapts to any nonlinear function defined by a representative set of training data. Once trained, the ANN model can remember a functional relationship and be used for further calculations. For these reasons, the ANN concept has been adopted to develop strongly nonlinear models for multiple sensors data fusion. Thomas *et al.* discussed the optimal fusion method of TV and infrared images using artificial neural networks [25]. After that, many neural network models have been proposed for image fusion such as BP, SOFM, and ARTMAP neural networks. BP algorithm has been mostly used. However, the convergence of BP networks is slow and the global minima of the error space may not be always achieved [26]. As an unsupervised network, SOFM network clusters input sample through competitive learning. But the number of output neurons should be set before constructing neural networks model [27]. RBF neural network can approximate objective function at any precise level if enough hidden units are provided. The advantages of RBF network training include no iteration, few training parameters, high training speed, simply process and memory functions [28]. Hong explored the way that using RBF neural networks combined with nearest neighbor clustering method to cluster, and membership weighting is used to fuse. Experiments show this method can obtain the better effect of cluster fusion with proper width parameter [29].

Gail *et al.* used Adaptive Resonance Theory (ART) neural networks to form a new framework for self-organizing information fusion. The ARTMAP neural network can act as a self-organizing expert system to derive hierarchical knowledge structures from inconsistent training data [30]. ARTMAP information fusion resolves apparent contradictions in input pixel labels by assigning output classes to levels in a knowledge hierarchy [31]. Rong *et al.* presented a feature-level image fusion method based on segmentation region and neural networks. The results indicated that this combined fusion scheme was more efficient than that of traditional methods [32].

The ANN-based fusion method exploits the pattern recognition capabilities of artificial neural networks, and meanwhile, the learning capability of neural networks makes it feasible to customize the image fusion process. Many of applications indicated that the ANN-based fusion methods had more advantages than traditional statistical methods, especially when input multiple sensor data were incomplete or with much noises. It is often served as an efficient decision level fusion tools for its self learning characters, especially in land use/land cover classification. In addition, the multiple inputs - multiple outputs framework make it to be an possible approach to fuse high dimension data, such as long-term time-series data or hyper-spectral data.

## 3. Advances in Applications

The goal of multiple sensor data fusion is to integrate complementary and redundant information to provide a composite image which could be used to better understanding of the entire scene. It has been widely used in many fields of remote sensing, such as object identification, classification, and change detection. The following paragraphs describe the recent achievements of image fusion in more detail.

### 3.1. Object identification

The feature enhancement capability of image fusion is visually apparent in VIR/VIR combinations that often results in images that are superior to the original data. In order to maximize the amount of information extracted from satellite image data useful products can be found in fused images [2]. A Dempster-Shafer fusion method for urban building detection was presented in 2004. First and last pulse of LIDAR data and multi-spectral aerial imagery were used. Apart from buildings, the classes “tree”, “grass land”, and “bare soil” are also distinguished by a classification method based on the Dempster-Shafer theory of data fusion. Identification of linear objects such as roads could also benefit from image fusion techniques. An integrated system for automatic road mapping from high-resolution multi-spectral satellite imagery by information fusion was discussed by Jin *et al*. in 2005 [33]. Andrea presents a solution to enhance the spatial resolution of MS images with high-resolution PAN data. The proposed method exploits the undecimated discrete wavelet transform, and the vector multi-scale Kalman filter, which is used to model the injection process of wavelet details. Fusion simulations on spatially degraded data and fusion tests at the full scale reveal that an accurate and reliable PAN-sharpening is achieved by the proposed method [34].

### 3.2. Classification

Classification is one of the key tasks of remote sensing applications. The classification accuracy of remote sensing images is improved when multiple source image data are introduced to the processing [2]. Images from microwave and optical sensors offer complementary information that helps in discriminating the different classes. As discussed in the work of Wang *et al.*, a multi-sensor decision level image fusion algorithm based on fuzzy theory are used for classification of each sensor image, and the classification results are fused by the fusion rule. Interesting result was achieved mainly for the high speed classification and efficient fusion of complementary information [35]. Land-use/land-cover classification had been improved using data fusion techniques such as ANN and the Dempster-Shafer theory of evidence. The Dempster-Shafer theory of evidence method uses a limited number of prototypes as items of evidence and can be implemented in a modified FKCN with specific architecture consisting of one input layer, a prototype layer, a combination and output layer, and decision layer. The experimental results show that the excellent performance of classification as compared to existing classification techniques [36,37].

### 3.3. Change detection

**Figure 4.**
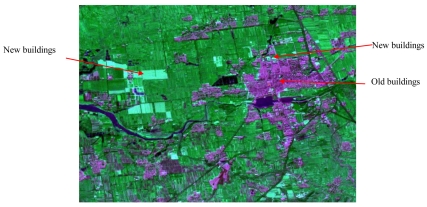
Image fusion for change detection: an example in Yanqing city, Beijing, China.

Change detection is the process of identifying differences in the state of an object or phenomenon by observing it at different times [38]. Change detection is an important process in monitoring and managing natural resources and urban development because it provides quantitative analysis of the spatial distribution of the population of interest [39]. Image fusion for change detection takes advantage of the different configurations of the platforms carrying the sensors. The combination of these temporal images in same place enhances information on changes that might have occurred in the area observed. Sensor image data with low temporal resolution and high spatial resolution can be fused with high temporal resolution data to enhance the changing information of certain ground objects. For example (Figure 4), Spot 5 Panchromatic band data with spatial resolution of 2.5 m of Yanqing city, Beijing China, in 2005 was fused with multiple spectral bands of Landsat TM data (spatial resolution: 30 m) in 2007. A simple Brovey transformation fusion method was used and the 3^rd^, 4^th^, 7^th^ bands of TM were selected for calculation. The building areas remained unchanged from 2005–2007 were grey-purple, meanwhile, the newly established buildings were highlighted (lime color in Figure 4) in the composed image and could be easily detected.

Madhavan *et al.* presented a decision level fusion system that automatically performs fusion of information from multi-spectral, multi-resolution, and multi-temporal high-resolution airborne data for a change-detection analysis. Changes are automatically detected in buildings, building structures, roofs, roof color, industrial structures, smaller vehicles, and vegetation [40]. In recent years, object-oriented processing techniques are becoming more popular, compared to traditional pixel-based image analysis, Object-oriented change information is necessary in decision support systems and uncertainty management strategies. An in-depth paper presented by Ruvimbo *et al.* introduced the concept and applications of Object-oriented change detection for urban areas [39]. In general, due to the extensive statistical and derived information available with the object-oriented approach, a number of change images can be presented depending on research objectives. In land use and land cover analysis; this level of precision is valuable as analysis at the object level enables linkage with other GIS databases or derived socio-economic attributes.

### 3.4. Maneuvering target tracking

Maneuvering target tracking is a fundamental task in intelligent vehicle research. With the development of sensor techniques and signal/image processing methods, automatic maneuvering targets tracking can be conducted operationally. Meanwhile, multi-sensor fusion is found to be a powerful tool to improve tracking efficiency. The tracking of objects using distributed multiple sensors is an important field of work in the application areas of autonomous robotics, military applications, and mobile systems [41].

The numbers of the papers focused on the problem of fusion between radar and image sensors in targets tracking have appeared in recent years [42,43]. Fusion of radar data and infrared images could improve the positioning accuracy and narrow down the image working area [43,44]. Vahdati-khajeh addressed the multi-target tracking problem for maneuvering targets in cluttered environments. The multiple scan joint probabilistic data association (MJPDA) algorithm was used for the sake of overcoming the problem of clutter points and targets which have joint observation [45]. In order to overcome the defects of the current statistical model on non-maneuvering target tracking, Chen *et al.* presented a novel multi-sensor data fusion algorithm for tracking the large-scale maneuvering target. The fuzzy adaptive Kalman filtering algorithm with maneuvering detection was used for large-scale maneuvering target which extracts feature data from Kalman filtering processes to estimate the magnitude and time of maneuvering. The simulation results showed that the tracking system with active and passive radar has higher precision than those with a single sensor for large-scale problems [42].

## 4. Discussion and Conclusions

Multi-sensor image fusion seeks to combine information from different images to obtain more inferences than can be derived from a single sensor. It is widely recognized as an efficient tool for improving overall performance in image based application. The paper provides a state-of-art of multi-sensor image fusion in the field of remote sensing. Below are some emerging challenges, along with recommendations building on the discussion in the previous sections.

### (1) Improvements of fusion algorithms

Among the hundreds of variations of image fusion techniques, the most popular and effective methods including IHS, PCA, Brovey transform, wavelet transform, and Artificial Neural Network (ANN). For convenient methods (e.g., HIS, PCA and Brovey transform), which have lower complexity and faster processing time, the most significant problem is color distortion [9]. Wavelet-based schemes perform better than convenient methods in terms of minimizing color distortion. The development of more sophisticated wavelet-based fusion algorithm (such as Ridgelet, Curvelet, and Contourlet transformation) could evidently improve performance result, but they often cause greater complexity in computation and parameters setting. Another challenge on existing fusion techniques will be the ability for processing hyper-spectral satellite sensor data. Artificial neural network seem to be one possible approach to handle the high dimension nature of hyper-spectral satellite sensor data.

### (2) Development of “algorithm fusion” methods

As mentioned above, each fusion method has its own set of advantages and limitations. The combination of several different fusion schemes has been approved to be the useful strategy which may achieve better quality of results [9,17]. As a case in point, quite a few researchers have focused on incorporating the traditional IHS method into wavelet transforms, since the IHS fusion method performs well spatially while the wavelet methods perform well spectrally [17,46]. However, selection and arrangement of those candidate fusion schemes are quite arbitrary and often depends upon the user's experience. Optimal combining strategy for different fusion algorithms, in another word, ‘algorithm fusion’ strategy, is thus urgent needed. Further investigations are necessary for the following aspects:

- Design of a general framework for combination of different fusion approaches;
- Development of new approaches which can combine aspects of pixel/feature/decision level image fusion;
- Establishment of automatic quality assessment method for evaluation of fusion results, which is discussed as follows.

### (3) Establishment of an automatic quality assessment scheme

Automatic quality assessment is highly desirable to evaluate the possible benefits of fusion, to determine an optimal setting of parameters for a certain fusion scheme, as well as to compare results obtained with different algorithms [17]. Mathematical methods were used to judge the quality of merged imagery in respect to their improvement of spatial resolution while preserving the spectral content of the data. Statistical indices, such as cross entropy, mean square error, signal-to-noise ratio, have been used for evaluation purpose. While recently a few image fusion quality measures have been proposed, analytical studies of these measures have been lacking. The work of Yin *et al.* focused on one popular mutual information-based quality measure and weighted averaging image fusion [47]. Jiying presented a new metric based on image phase congruency to assess the performance of the image fusion algorithm [48]. However, in general, no automatic solution has been achieved to consistently produce high quality fusion for different data sets [49]. It is expected that the result of fusing data from multiple independent sensors will offer the potential for better performance than can be achieved by either sensor, and will reduce vulnerability to sensor specific countermeasures and deployment factors. We expect that future research will address new performance assessment criteria and automatic quality assessment methods.
